# Clinical and Histopathological Amelioration of Experimental Autoimmune Encephalomyelitis by AAV Vectors Expressing a Soluble Interleukin-23 Receptor

**DOI:** 10.1007/s13311-017-0545-8

**Published:** 2017-06-07

**Authors:** Marta Miralles, Herena Eixarch, Marcos Tejero, Carme Costa, Keiji Hirota, A. Raul Castaño, Meritxell Puig, Gitta Stockinger, Xavier Montalban, Assumpció Bosch, Carmen Espejo, Miguel Chillon

**Affiliations:** 1 Institut de Neurociències (INc), Departament Bioquímica i Biologia Molecular, Universitat Autònoma Barcelona, Bellaterra, Spain; 2 Servei de Neurologia-Neuroimmunologia, Centre d’Esclerosi Múltiple de Catalunya, Vall d’Hebron Institut de Recerca, Hospital Universitari Vall d’Hebron, Barcelona, Spain; 3 Universitat Autònoma de Barcelona, Bellaterra, Cerdanyola del Vallès 08193 Spain; 4 MRC National Institute for Medical Research, London, UK; 5 IBB, Departament Biología Celular, de Fisiología y de Immunología, Universitat Autònoma Barcelona, Bellaterra, Spain; 6 Centro de Investigación Biomédica en Red sobre Enfermedades Neurodegenerativas (CIBERNED), Instituto de Salud Carlos III, Madrid, Spain; 7 Institució Catalana de Recerca i Estudis Avançats (ICREA), Barcelona, Spain; 8 Vall d’Hebron Institut de Recerca, Hospital Universitari Vall d’Hebron, Barcelona, Spain; 9 Vector Production Unit (UPV), Universitat Autònoma Barcelona, Barcelona, Spain

**Keywords:** Multiple sclerosis, IL-23R, Th17, EAE, AAV vector

## Abstract

The role of the T helper (Th)17 pathway has been clearly demonstrated in the onset and progression of autoimmune diseases, where interleukin (IL)-23 is a key molecule in maintaining the response mediated by Th17 cells. As a consequence, recent strategies based on blocking the interaction between IL-23 and its receptor (IL-23R), for example the anti-p19 antibody tildrakizumab, have been developed to regulate the Th17 pathway from the initial stages of the disease. Here, a soluble (s)IL-23R cDNA was cloned in expression plasmids and viral vectors. The clinical efficacy of sIL-23R was evaluated in myelin oligodendrocyte glycoprotein-induced experimental autoimmune encephalomyelitis mice intravenously injected with a single dose of adeno-associated virus AAV8–sIL-23R vectors. Cytokine secretion was determined by multiplex assay, while histopathological analysis of the central nervous system was performed to study demyelination, inflammatory infiltration, and microglia and astroglia activation. We observed that administration of adeno-associated vector 8 encoding sIL-23R was associated with a significant disease improvement, including delay in the onset of the clinical signs; slower progress of the disease; interference with IL-23-mediated signal transducer and activator of transcription response by inhibiting of signal transducer and activator of transcription 3 phosphorylation; reduced demyelination and infiltration in the central nervous system; and lower astrocyte and microglia activation. Our results suggest that the use of vectors carrying sIL-23R to block the IL-23/IL-23R interaction may be a new therapeutic strategy for the treatment of multiple sclerosis.

**Electronic supplementary material:**

The online version of this article (doi:10.1007/s13311-017-0545-8) contains supplementary material, which is available to authorized users.

## Introduction

Multiple sclerosis (MS) is a chronic neurodegenerative and demyelinating autoimmune disease that affects an estimated 2.5 million people worldwide. Unfortunately, despite efforts to develop new therapies, there is no curative treatment, and long-term prognosis remains poor for patients.

Recently, the role of the T helper (Th)17 pathway has been demonstrated in the development of MS, where interleukin (IL)-23 is a key molecule in maintaining the response mediated by Th17 cells [1–3]. Compared with healthy controls, 40% of patients with MS have an increased number of Th17 cells in the peripheral blood [4]. In addition, patients in the acute phase of the disease had 3.5 times more Th17 cells than patients in remission. Furthermore, patients with MS also had more Th17 cells in the cerebrospinal fluid than patients with noninflammatory neurological diseases, suggesting their recruitment or enrichment to sites of inflammation in MS [5]. It is noteworthy that adoptive transfer of myelin oligodendrocyte glycoprotein (MOG)-specific Th17 cells reproduces experimental autoimmune encephalomyelitis (EAE), an experimental model of MS. Autoantigen-specific Th17 cells cross the blood–brain barrier and infiltrate the central nervous system (CNS) before the clinical signs of EAE [6]. Furthermore, the administration of anti-IL-17 and anti-IL-23 monoclonal antibodies in an EAE model reduced the severity of the disease [7–10]. Recent data suggest that IL-23 is not only necessary to stabilize the population of Th17 cells in the secondary lymphoid tissue, but also to maintain a pathogenic Th17 population at the site of inflammation [11]. Although IL-23p19-deficient mice were resistant to EAE [1], IL-17-deficient mice developed the disease [12, 13]. Thus, inhibition of IL-23 may be a strategy to control the deregulation of the Th17 pathway during the initial stages of the disease and the evolution of the inflammatory autoimmune response.

Different immunosuppressors have been used for MS in animals and in human clinical trials. For example, ustekinumab is a monoclonal antibody against p40, the common subunit of IL-12 and IL-23, and is effective against psoriasis and Crohn's disease [14, 15] but has not succeeded in MS [16], possibly because it is directed against the p40 domain and therefore not only inhibits IL-23, but also IL-12. As an alternative, specific biological treatments for the p19 subunit of the IL-23 heterodimer have been developed. Interestingly, a recent study performed by Guo et al. [17] shows that the continuous administration of soluble recombinant IL-23 receptor (IL-23R) cytokine-binding homology region blocks the interaction between IL-23 and the IL-23R and ameliorates the clinical signs of EAE, paving the way for similar strategies, such as secukinumab (anti-IL-17A antibody), to treat Th17-mediated autoimmune diseases, including MS [18].

For the purpose of finding new strategies in the fight against MS based on the control of the Th17 inflammatory axis, here we report that a single administration of adeno-associated virus (AAV) vectors carrying the coding sequence of soluble IL-23R (sIL-23R) delays the onset of the clinical signs, slows the progress of the disease, and reduces the inflammatory infiltration and demyelination in the CNS in a MOG-induced EAE mouse model.

## Methods

### Viral Vector Generation, Production, and Purification

AAV8 and first-generation Adenovirus 5 (Ad5) vectors were produced, purified, and manipulated at biosafety level 2 facilities at the Universitat Autònoma Barcelona, Spain. Briefly, for the production of Ad5 vectors, *Pac*I-linearized plasmids (6 μg) containing the adenovirus genomes, as well as green fluorescent protein (GFP), mouse IL-23R, sIL-23R, or null sequences, were transfected into 1 × 10^6^ HEK293 cells, and the virus was recovered 8 to 10 days post-transfection. Then, viruses were sequentially amplified until the infection of 4 × 10^8^ HEK293 cells. Viral particles were measured by absorbance of disrupted virions at 260 nm where one OD equals 1 × 10^12^ particles/ml, while infective particles were measured by an endpoint dilution assay that counted the number of hexon-producing cells in triplicate [19, 20]. AAV8 vectors were generated using the triple-transfection system in HEK293 cells. After 48 h, AAV vectors were harvested, treated with benzonase, purified in an iodixanol gradient, and titrated using the PicoGreen® system [21]. In all cases, transgene expression was driven by a cytomegalovirus promoter.

### Immunodetection and Immunoprecipitation of sIL-23R

HEK293 and Jurkat cells were sonicated and homogenized in lysis buffer [50 mM Tris-Cl (pH 7.4), 150 mM NaCl, 1 mM ethylenediamine tetraacetic acid, 1% NP-40, 0.25% sodium deoxycholate and Protease Inhibitor Cocktail Set I (Millipore, Billerica, MA, USA)]. Total protein concentration was determined using the Pierce BCA Protein Assay (Thermo Fisher Scientific, Waltham, MA, USA) according to the manufacturer’s instructions and with bovine serum albumin as the standard. Sample absorbance at 562 nm was measured using a NanoDrop 1000 UV/Vis spectrophotometer (Thermo Fisher Scientific). Protein extracts (15–25 μg/sample) were loaded onto denaturing acrylamide gels and then electrotransferred to polyvinylidene fluoride membranes (Amersham, Little Chalfont, UK). Primary antibodies were incubated in the presence of 5% (w/v) skimmed milk combined with Western blotting detection reagent (ECL Plus; Amersham). Band pixel intensities were quantified using Image J (National Institutes of Health, Bethesda, MD, USA) and normalized by antiactin levels in each lane. Anti-IL-23R (AF1686; R&D Systems, Minneapolis, MN, USA) and secondary antibody rabbit antigoat Ig horseradish peroxidase (P0160; Dako, Glostrup, Denmark) were used at a dilution of 1:2000 and 1:5000, respectively. Antioctaprobe (Sc-807; Santa Cruz Biotechnology, Santa Cruz, CA, USA) and secondary antibody swine antirabbit Ig horseradish peroxidase (P0399; Dako) were used at a dilution of 1:500 and 1:5000, respectively.

HEK293 cells transfected with the GST–sIL-23R or the sIL-23R plasmids were collected and proteins (extracted as described previously) were incubated with glutathione-sepharose 4B (GE Healthcare Life Sciences, Uppsala, Sweden) at 4°C for 2 h. Samples were rinsed 3 times, and eluted with glutathione-eluted buffer [50 mM Tris-HCl, 0.1 mM ethylene glycol-bis (β-animoethyl ether)-N,N,N',N'-tetraacetic acid, 0.275 M saccharose, 0.1% β-mercaptoethanol, 40 mM reduced glutathione, pH 8.0] for 25 min with agitation. Finally, samples were incubated at room temperature for 5 min, and supernatants were transferred to fresh tubes and stored at –80°C until use.

### Western Analysis

sIL-23R was produced in 150-mm plates (HEK-293 cells in Dulbecco’s modified eagle medium + 1% fetal bovine serum) and infected with Ad5/sIL-23R or control Ad5/GFP at a multiplicity of infection of 10. Cells were harvested 28 h postinfection, lysed, and crude lysates stored at –80°C until use. Splenocytes (2 × 10^6^ cells/ml, 3 ml of final volume) were cultured in 6-well plates with sIL-23R-conditioned medium. One h after seeding, IL-23 (final concentration of 10 ng/ml) was added to cells for 5 min. In competition experiments, IL-23 was previously co-incubated with sIL-23R-conditioned medium for 1 h. Splenocytes were harvested, medium discarded by centrifugation, and cells resuspended in 100 μl lysis buffer (25 mM Tris-HCl at pH 7.6, 210 mM NaCl, 1 mM ethylenediaminetetraacetic acid, 1% Nonidet P40, 0.1% sodium dodecyl sulfate) containing a mixture of protease and phosphatase inhibitors (Millipore, Darmstadt, Germany). Antibodies against total signal transducer and activator of transcription (STAT)3, and Y705-phosphoryl STAT3 (pY705-STAT3), were from Cell Signaling Technology (Danvers, MA, USA), and antibody against actin from Sigma Chemicals (St. Louis, MO, USA). Cell debris was removed by centrifugation. Protein (40 μg) was loaded onto 10% (wt/vol) sodium dodecyl sulfate polyacrylamide electrophoresis gels. The separated proteins were transferred to polyvinylidene fluoride membranes (Millipore, Darmstadt, Germany). The membranes were incubated with primary antibody at 4°C overnight, followed by incubation with secondary antibody for 1 h at room temperature. Band intensity was quantified using ImageJ.

### β-Galactosidase Analysis

To facilitate quantitation of transgene expression an AAV8 vector expressing β-galactosidase was used. AAV8 serotype was selected owing to its high efficiency in infecting several organs, including liver and skeletal muscle, after intravenous administration, and also because AAV8-mediated transgene expression is stable for years. Animals injected with AAV8–β-galactosidase [5 × 10^11^ viral genomes (vg)/mouse; *n* = 4 or 5 per group] were euthanized 3 weeks after AAV administration, so all animals were expressing β-galactosidase the same period of time. *In vivo*, β-galactosidase expression was quantified using the Galacto-Light Plus System (Applied Biosystems, Foster City, CA, USA) and a luminometer (Monolight 2010; Analytical Luminescence Laboratory, USA) according to the manufacturers’ recommendations. β-Galactosidase activity was normalized to total protein concentration (protein assay reagent; Bio-Rad Laboratories, Hercules, CA, USA).

### Animals

C57BL/6J 8–10-week-old female mice purchased from Harlan Laboratories (Milan, Italy) were used. Mice were housed under standard light- and climate-controlled conditions, and standard chow and water were provided *ad libitum*.

### Ethics, Consent, and Permissions

All experiments were performed in strict accordance with European Union and governmental regulations (Generalitat de Catalunya Decret 214/97 30 July). The Ethics Committee on Animal Experimentation of the Vall d’Hebron Research Institute approved all procedures described in this study (protocol number: CEEA 40/10/11-DAAM 5614; CEEA 81/11-DAAM 6364).

### *In Vivo* Administration of AAV Vectors

In order to define an optimal time of vector administration, a preliminary *in vivo* experiment was performed before and after EAE induction. To facilitate quantitation of transgene expression an AAV8 vector expressing β-galactosidase was used. AAV8 serotype was selected owing to its high efficiency in infecting several organs, including liver and skeletal muscle, after intravenous administration, and also because AAV8-mediated transgene expression is stable for years. AAV8–β-galactosidase vectors (5 × 10^11^ vg/mouse; *n* = 4 or 5 per group) were injected at days –6, –2, +5, and +9 postimmunization (p.i.), and euthanized 3 weeks after AAV administration; therefore, all animals were expressing β-galactosidase in the same period of time.

To analyze the effect of sIL-23R expression in EAE outcome, 18 days before EAE induction, a single dose (9 × 10^10^–5 × 10^11^ vg/mouse) of AAV8 vectors (sIL-23R or null) was administered by intravenous injection through the lateral tail vein.

### EAE Induction and Clinical Follow-Up

Anesthetized mice were immunized by subcutaneous injections of 100 μl phosphate-buffered saline (PBS) containing 100 μg MOG peptide 40–55 (MOG_40–55_) (Proteomics Section, Universitat Pompeu Fabra, Barcelona, Spain) emulsified in 100 μl Complete Freund's Adjuvant (Sigma Chemicals) containing 4 mg/ml *Mycobacterium tuberculosis* H37RA (Difco Laboratories, Franklin Lakes, NJ, USA). At days 0 and 2 p.i., mice were intravenously injected with 250 ng pertussis toxin (Sigma Chemicals). Two animals per group were used as control mice (sham immunization), which were immunized in the same manner using PBS in the absence of the peptide. Mice were weighed and examined daily for neurological signs using the following criteria: 0 = no clinical signs; 0.5 = partial loss of tail tonus for 2 consecutive days; 1 = paralysis of whole tail; 2 = mild paraparesis of one or both hindlimbs; 2.5 = severe paraparesis or paraplegia; 3 = mild tetraparesis; 4 = tetraparesis (severe in hindlimbs); 4.5 = severe tetraparesis 5 = tetraplegia; 6 = death (modified from [22]). All data presented are in accordance with the guidelines suggested for EAE publication [23]. Weight loss was calculated as the percentage change in daily weight compared with the initial weight on the day of immunization. Score 5 and weight loss > 30% were defined as endpoint criteria to minimize suffering and guarantee animal welfare. In the different experiments, incidence of EAE in the EAE control untreated group (*n* = 8-13 per group in each experiment) was 100%.

### Splenocyte Proliferative Assay and Cytokine Production

Five mice per group were used to perform immunological assays. Splenocytes were removed from euthanized mice at day 14 p.i. and seeded at 2 × 10^5^ cells/well in a 96-well plates in Iscove’s modified Dulbecco’s medium (PAA Laboratories GmbH, Pasching, Austria) supplemented with 10% HyClone FetalCloneI (Thermo Fisher Scientific), 50 μmol/l 2-mercaptoethanol (Sigma Chemicals), 2 mmol/l L-glutamine, 50 U/ml penicillin, and 50 mg/ml streptomycin, all obtained from Gibco (Paisley, UK), and 5 μg/ml MOG_40–55_ or 5 μg/ml phytohemagglutinin (PHA) (Sigma Chemicals). Cells cultured without any stimulus were used as controls. After 48 h, the supernatants (50 μl/well) were harvested and stored at –80°C to further assess cytokine release. Then, the cell cultures were incubated in the presence of 1 μCi/well [3H]-thymidine (Perkin Elmer, Waltham, MA, USA) under the same conditions for an additional 18 h, and the levels of incorporated radioactivity were measured using a β-scintillation counter (Wallac, Turku, Finland). Five replicates for each mouse and condition were performed. The stimulation index was calculated by dividing the arithmetic mean of counts per minute from stimulated cultures by the arithmetic mean of counts per minute from control cultures.

Cytokine secretion was determined both in the supernatants of MOG_40–55_-stimulated splenocytes and in the serum of 5 mice per group by flow cytometry using the MILLIPLEX MAP Mouse Th17 Magnetic Bead Panel-Immunology Multiplex Assay (Merck-Millipore, Darmstadt, Germany), according to the manufacturer’s instructions. Quantification was performed using Luminex MagPix and the xPONENT 4.2 software.

### Histopathological Studies

At day 14 p.i., 5 mice from each group were euthanized and brains and spinal cords were collected and fixed in 4% paraformaldehyde at 4°C for 48 h. Samples were washed with PBS and incubated in 30% saccharose at 4°C for cryoprotection. Then, samples were embedded in OCT, frozen on dry ice, and cryosectioned into 20-μm slices. To determine the inflammatory infiltrates hematoxylin and eosin staining was performed, and demyelination was assessed by Klüver–Barrera staining. Cell infiltration was evaluated using hematoxylin and eosin staining according to the following criteria: 0 = no lesion; 1 = cellular infiltration only in the meninges; 2 = very discrete and superficial infiltrates in the parenchyma; 3 = moderate infiltrate (< 25%) in the white matter; 4 = severe infiltrates (< 50%) in the white matter; 5 = more severe infiltrates (> 50%) in the white matter. Demyelination (Klüver–Barrera staining) was scored as follows: 0 = no demyelination; 1 = little demyelination, only around infiltrates, and involving < 25% of the white matter; 2 = demyelination involving < 50% of the white matter; 3 = diffuse and widespread demyelination involving > 50% of the white matter.

Microglia and astroglia activation was analyzed using a Leica TCS SP2 confocal microscope by quantifying the integrated fluorescence intensity of specific markers Iba1 (Wako-019-19741; WAKO, Osaka, Japan) for microglia, and GFAP (Sigma-G3893; Sigma Chemicals) for astroglia using the Metamorph 5.0r1 program.

### Statistical Analysis

Data are expressed as the mean ± SEM values unless otherwise stated. Statistical analysis was performed using G-Stat version 2.0 and Prism 5.04 software (GraphPad Inc., La Jolla, CA, USA). Except for the results presented in Figs 3B and 4B, statistical significance between individual groups was determined by nonparametric Mann–Whitney or Kruskal–Wallis tests. Analysis of variance plus post-hoc Tukey’s honest significant difference test was used for the results shown in Fig. 3B, while a *t*-test (unpaired, 2-tailed) was used for the results shown in Fig. 4B. In all the statistical analyses, *p* < 0.05 was considered significant.

## Results

### Design and Cloning of Murine sIL-23R

**Fig. 1:**
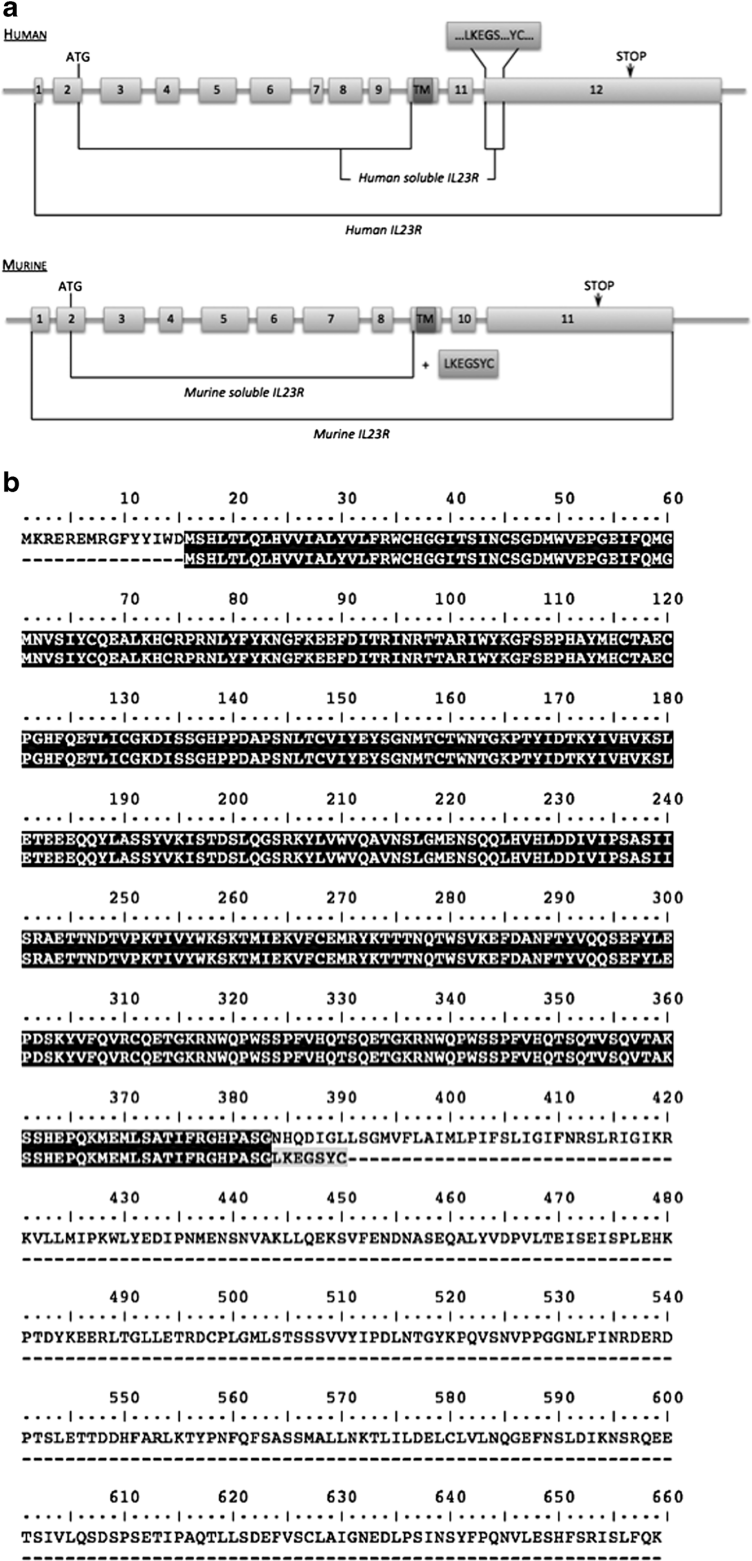
(A) Comparison of the human and murine interleukin-23 receptor (IL-23R) genomic structures and the design scheme based on the soluble human (s)IL-23R isoform. (B) Amino-acid sequence of murine IL-23R and designed murine sIL-23R. Amino acids in blue were added by analogy with soluble human IL-23R

Human sIL-23R may be naturally produced by alternative splicing to generate a sIL-23R isoform [24]. However, the soluble form of the murine IL-23R (NCBI GenBank NM_144548.1) has not yet been described. We designed the equivalent mouse sIL-23R by mimicking the alternative splicing of the human sIL-23R that essentially eliminates exons 10 and 12. First, by analogy to human IL-23R4, we selected the sequence between the initiation ATG codon of exon 2 and the beginning of exon 10, just before the sequence corresponding to the transmembrane region (Fig. 1A). Furthermore, since the C-terminus of human sIL-23R includes a set of amino acids (LKEGSYC) belonging to exon 12, the same amino-acid sequence was also added to murine sIL-23R in case it was important for the function or stability of the protein (Fig. 1B). According to protein prediction programs, the designed sIL-23R gene should be secreted extracellularly. Murine sIL-23R was generated by DNA synthesis, cloned into adenovirus vector (Ad)5 and AAV8 genomes, and its identity was confirmed by sequencing. Before *in vivo* experiment, AAV vector infectivity and AAV-mediated transgene expression were verified by *in vitro* infection and further mRNA analysis (data not shown).

### Cellular Distribution of IL-23R and sIL-23R

**Fig. 2:**
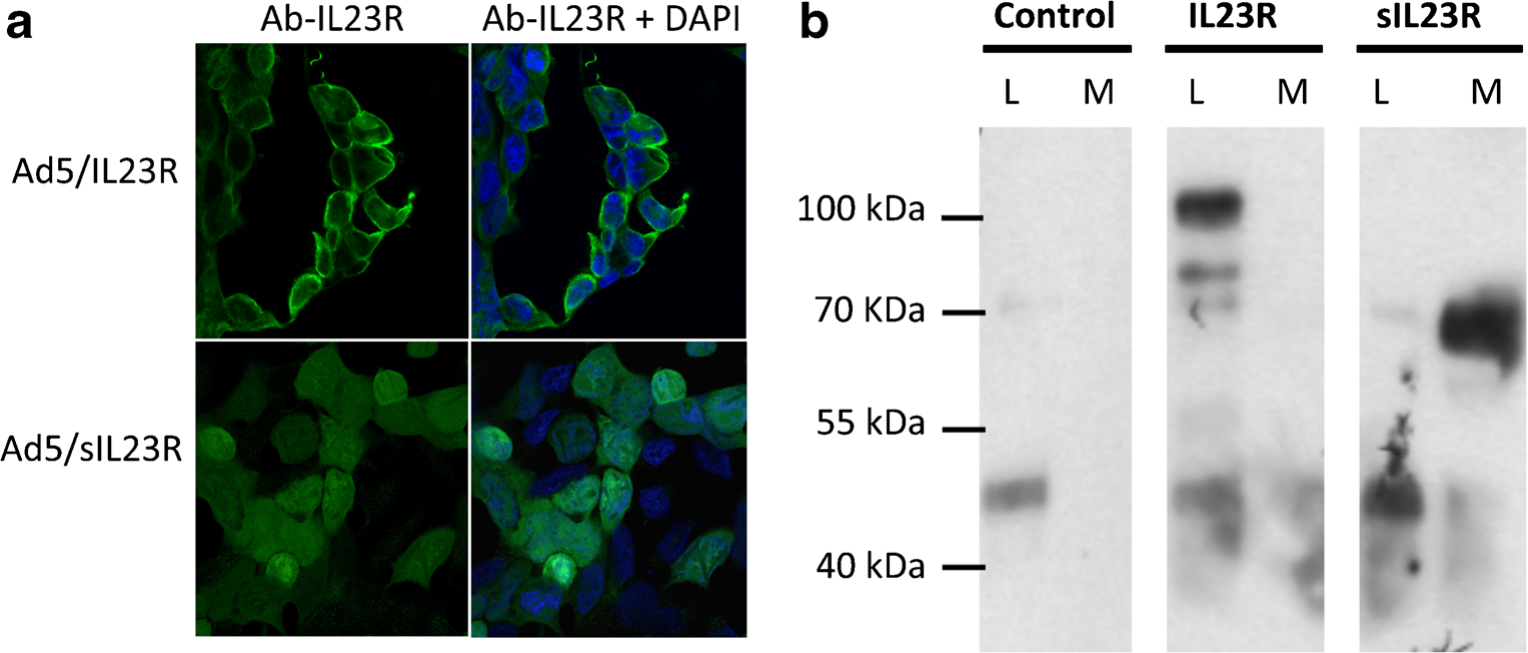
(A) Cellular distribution of interleukin-23 receptor (IL-23R) and soluble (s)IL-23R. Immunofluorescence with anti-IL-23R antibody (green). Nuclei are stained with 4,6-diamidino-2-phenylindole (DAPI; blue). Representative photos from 3 independent experiments (*n* = 5 wells each) where HEK293 cells were infected with adenovirus (Ad)5-IL-23R or Ad5–sIL-23R. (B) sIL-23R is produced and secreted into the extracellular medium. Western blotting with primary antioctaprobe antibody after immunoprecipitation. Samples: extracellular medium of Jurkat cells infected with Ad5–green fluorescent protein (control); Ad5–FLAG–IL-23R (IL-23R) or Ad5–FLAG–sIL-23R (sIL-23R). Experiment was performed 3 times, and representative results selected L = protein extract from cell lysates; M = protein extract from cell media

HEK293 cells were infected with Ad5–IL-23R, Ad5–FLAG–IL-23R, Ad5–sIL-23R, and Ad5–FLAG–sIL-23R with a multiplicity of infection of 2 in order to analyze the cellular distribution of IL-23R and sIL-23R. Two days after infection, the cells were fixed and analyzed by immunofluorescence with antibodies against IL-23R. Confocal microscopy showed that IL-23R was located in the membrane (Fig. 2A). These results were reproduced using the anti-FLAG and antioctaprobe antibodies (data not shown). In contrast, sIL-23R receptor was not found in the cell membrane but was diffuse in the cytoplasm (Fig. 2A).

To study whether sIL-23R was secreted or stayed within the cytoplasm, we measured the presence of sIL-23R in the culture medium. During our initial experiments we did not succeed in detecting murine sIL-23R using commercial anti-IL-23R antibodies, and so a new set of viral vectors, including either a FLAG-tag or a GST-tag at the N-terminus of the construct were generated. In addition, owing to the higher efficiency of Ad5 vectors compared with AAV8 vectors, to infect cells *in vitro*, we used Ad5 vectors for *in vitro* assays. To confirm the secretion of sIL-23R into the extracellular medium, Jurkat cells were infected with Ad5–sIL23R, and 2 days later, supernatants were concentrated and immunoprecipitated with anti-FLAG. As seen in Figure 2B, Western blotting with an antioctaprobe antibody showed a band of the expected size in protein extracts from cell media only when the sIL-23R–FLAG construct was used, while no band was detected when using the GFP or the transmembrane IL-23R controls.

### sIL-23R Inhibits IL-23R Signaling

**Fig. 3:**
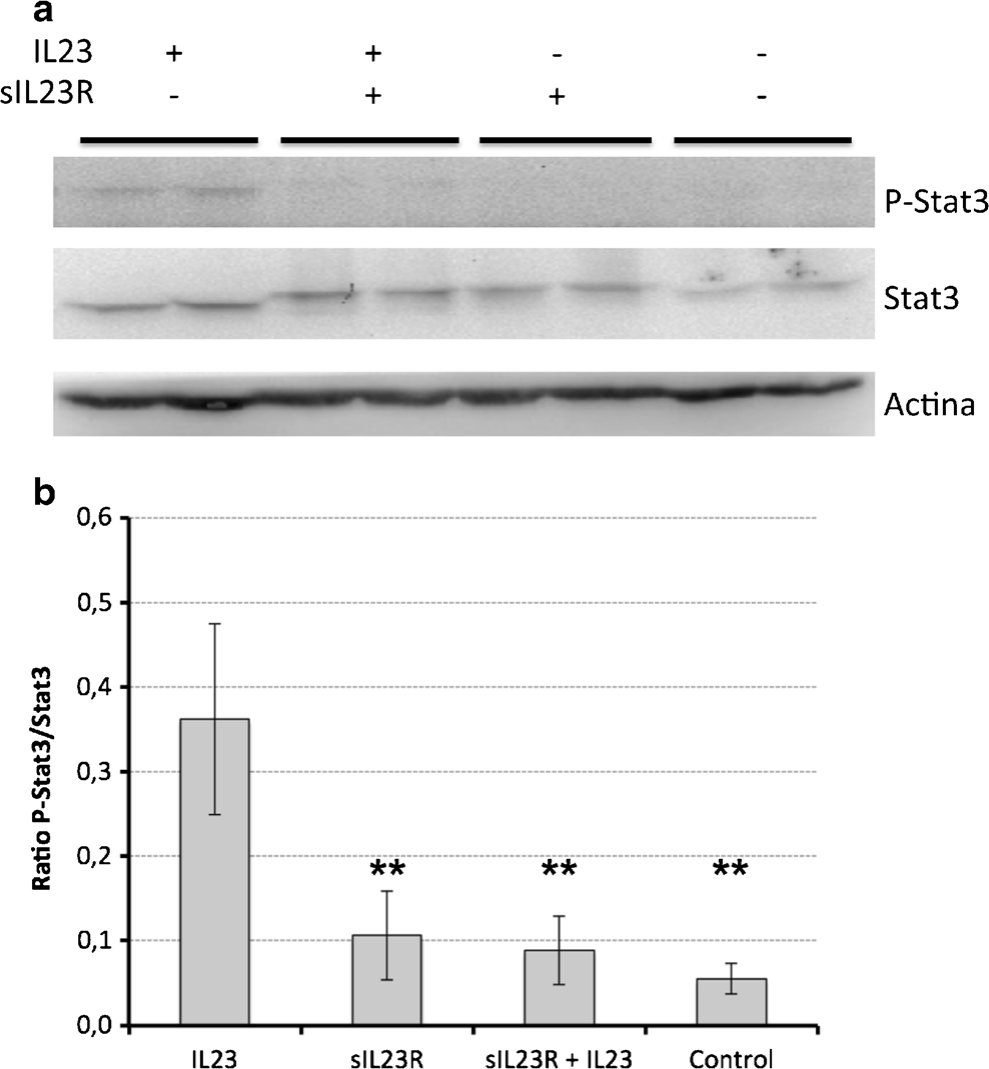
Soluble interleukin-23 receptor (sIL-23R)/interleukin-23 (IL-23) binding inhibits phosphorylation of signal transducer and activator of transcription 3 (STAT3) caused by interleukin (IL)-23. Splenocytes were incubated with 10 ng/ml of IL-23 for 5 min. When applied, IL-23 and sIL-23R were co-incubated for 1 h prior addition to cell culture media. (A) Representative Western blot for STAT3 and phosphorylated (p)STAT3. (B) Quantification of the ratio pSTAT3/STAT3 from 4 independent experiments (*n* = 2 or 3 per experiment). **p<0.01

Yu and Gallagher [25] have reported that the soluble variant of the human IL-23R binds IL-23 in solution (and not IL-12). This sIL-23R/IL-23 binding inhibits phosphorylation of STAT3 caused by IL-23 and subsequently, modulates the Th17 cells by inhibiting the production of the Th17-associated cytokines IL-17A and IL-17F. In order to know whether murine sIL-23R antagonize IL-23-mediated STAT3 response, splenocytes incubated with IL-23 and IL-23/sIL-23R were analyzed. As seen in Figure 3, co-incubation of IL-23 and sIL-23R does not alter levels of total STAT3 but abolishes STAT3 phosphorylation (*p* > 0.01), confirming the therapeutic potential of sIL-23R to functionally antagonize IL-23, and therefore to modulate the Th17 pathway.

### *In vivo* Administration of sIL-23R EAE Mice

**Fig. 4:**
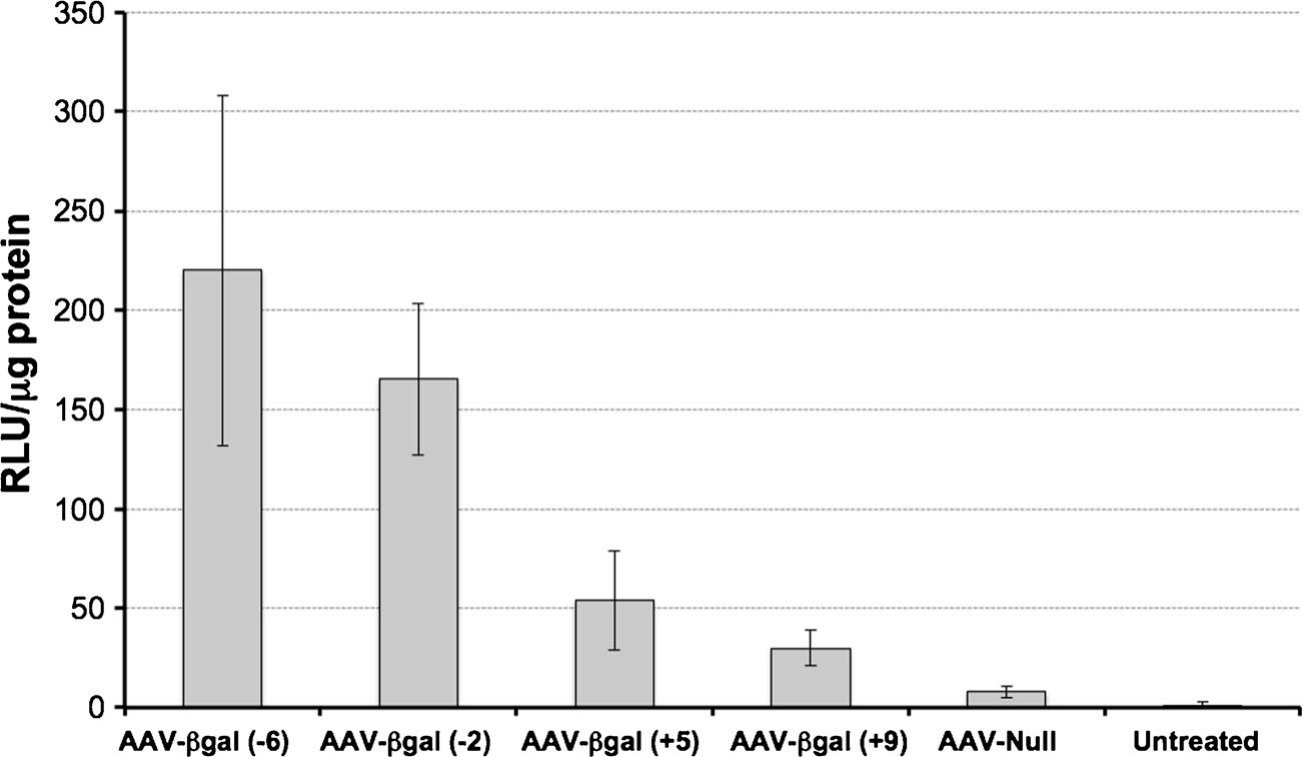
Adeno-associated virus AAV8-mediated β-galactosidase (βGal) expression before (day –6; day –2) and after (day +5; day +9) the induction of experimental autoimmune encephalomyelitis (EAE). Animals were untreated (EAE), or treated with 5 × 10^11^ viral genomes of AAV8-null or AAV8-βGal vectors. EAE induction is considered day +0. βGal expression was measured by relative light units (RLU) per μg of protein

In order to define an optimal time of vector administration, AAV8–β-galactosidase vectors (5 × 10^11^ vg/mouse; *n* = 4 or 5 per group) were injected at days –6, –2, +5, and +9 p.i., and euthanized 3 weeks after AAV administration. Interestingly, as seen in Figure 4, AAV8-mediated β-galactosidase expression was much higher when administering the vectors before EAE induction, while transgene expression was severely reduced when administering after EAE induction (between 3–4-fold and 6–8-fold higher than those observed at day +5 and day +9, respectively). This effect was more evident when vectors were administered 2 or 3 weeks before EAE induction (data not shown). Therefore, in this model for EAE only the preventive approach but not the therapeutic approach seems feasible, unless other strategies such as promoters that can be regulated can be used.

**Fig. 5:**
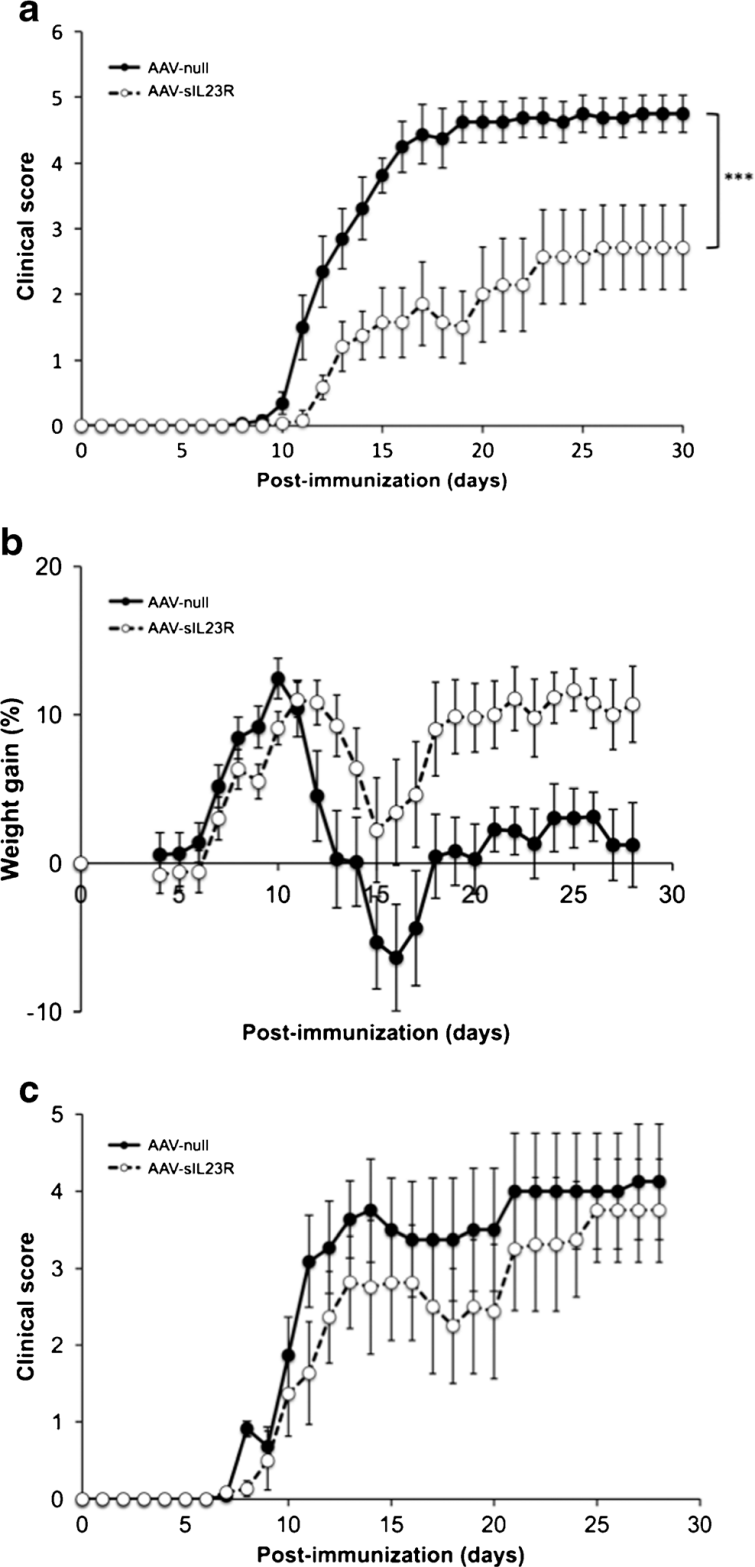
Administration of adeno-associated virus AAV8–soluble interleukin-23 receptor (sIL-23R) significantly reduces the clinical course of experimental autoimmune encephalomyelitis (EAE). (A) Mean daily clinical score for each group. Bars represent the SEM. The AAV8–sIL-23R group shows a statistically significant improvement in the clinical course of the disease compared with the control group treated with AAV8-null (*p* = 0.003). (B) Average daily weight change relative to initial weights on the day of EAE induction. Bars represent the SEM. Animals immunized with myelin oligodendrocytes glycoprotein (MOG) peptide 40–55 (MOG_40–55_) and treated with 5 × 10^11^ viral genome (vg)/mouse of AAV8–sIL-23R or AAV8-null (*n* = 8–13). (C) Lower dose of AAV8–sIL-23R did not improve the clinical course of EAE. The graph represents the mean daily clinical evaluation for each group. Bars represent the SEM. Animals immunized with MOG_40–55_ and treated with 9 × 10^10^ vg/mouse of AAV8–sIL-23R or AAV8-null (*n* = 8–11). ***p<0.001.

To analyze the effect of sIL-23R expression in EAE outcome, a dose of 5 10^11^ vg of AAV8–sIL-23R or AAV8-null was administered intravenously to each animal at day 18 preimmunization. As seen in Figure 5A, the animals treated with AAV8–sIL-23R showed a significant improvement in the clinical signs of the disease compared with those treated with the null vector. Moreover, clinical improvement was stable and sustained over time until the end of the experiment, with a mean clinical score on the last day of 4.69 ± 0.84 in the control group and of 2.71 ± 1.70 in the sIL-23R group (*p* = 0.012). In addition, the cumulative clinical score was 39.8 ± 28.2 for AAV8–sIL-23R-treated animals, while for the AAV8-null group it was 84.4 ± 12.9 (*p* = 0.003). Furthermore a trend toward a delay in disease onset was observed: 12.7 ± 2.6 for sIL-23R-treated mice, and 10.9 ± 1.4 for control-treated mice (*p* = 0.060). It is broadly described that EAE disease is associated with a body weight loss that may plateau during the chronic stage. We also observed that animals treated with the null virus had a more pronounced tendency towards weight loss than the group treated with sIL-23R during the experiment (*p* = 0.060; Fig. 5B).

Interestingly, the effect observed was dose-dependent, since lower doses (9 × 10^10^ vg/mouse) of the AAV8–sIL-23R vector only slightly ameliorated clinical signs without reaching statistical significance (mean cumulative score of 55.2 ± 19.2 for AAV8–sIL-23R-treated animals and 70.1 ± 19.3 for AAV8-null–treated animals; Fig. 5C).

### Immunological Analysis of Mice Treated with AAV8-sIL-23R

Five mice for each treatment condition were euthanized at 14 days p.i. for immunological and histopathological studies. Splenocytes from these mice were cultured in the presence of the MOG_40–55_ peptide or PHA for cell proliferation assays. Despite the differences observed in the clinical score, no differences were observed in the polyclonal proliferation to PHA of splenocytes between the null and sIL-23R groups (stimulation index of 44.8 ± 8.8 and 31.3 ± 3.7, respectively; *p* = 0.152), or the antigen-specific proliferation (stimulation index of 25.3 ± 2.5 and 28.1 ± 3.0, respectively; *p* = 0.457) (data not shown).

Next, the cytokines secreted into the culture medium of MOG_40–55_-stimulated splenocytes were analyzed using the Luminex system Magpix. The analysis showed a statistically significant difference in the concentration of interferon (IFN)-γ (null: 1765.9 ± 1146.1 pg/ml; sIL23R: 5691.3 ± 2639.3 pg/ml; *p* = 0.016), as well as a trend toward higher production of granulocyte macrophage colony-stimulating factor (null: 146.2 ± 106.6 pg/ml; sIL23R: 337.4 ± 188.0 pg/ml; *p* = 0.083), which did not reach statistical significance. For other cytokines (IL-2, IL-4, IL-5, IL-6, IL-10, IL-17A, IL-22), no differences were detected between the 2 groups. IL-21 was below the detection limit of the kit in all samples. In addition, cytokine concentrations in the serum were also analyzed. Only IFN-γ, IL-6, IL-5, and IL-22 reached the detection limit of the assay. However, none of these cytokines showed differences in serum concentration between the treated and control groups.

### Histopathological Analysis of the CNS

**Fig. 6:**
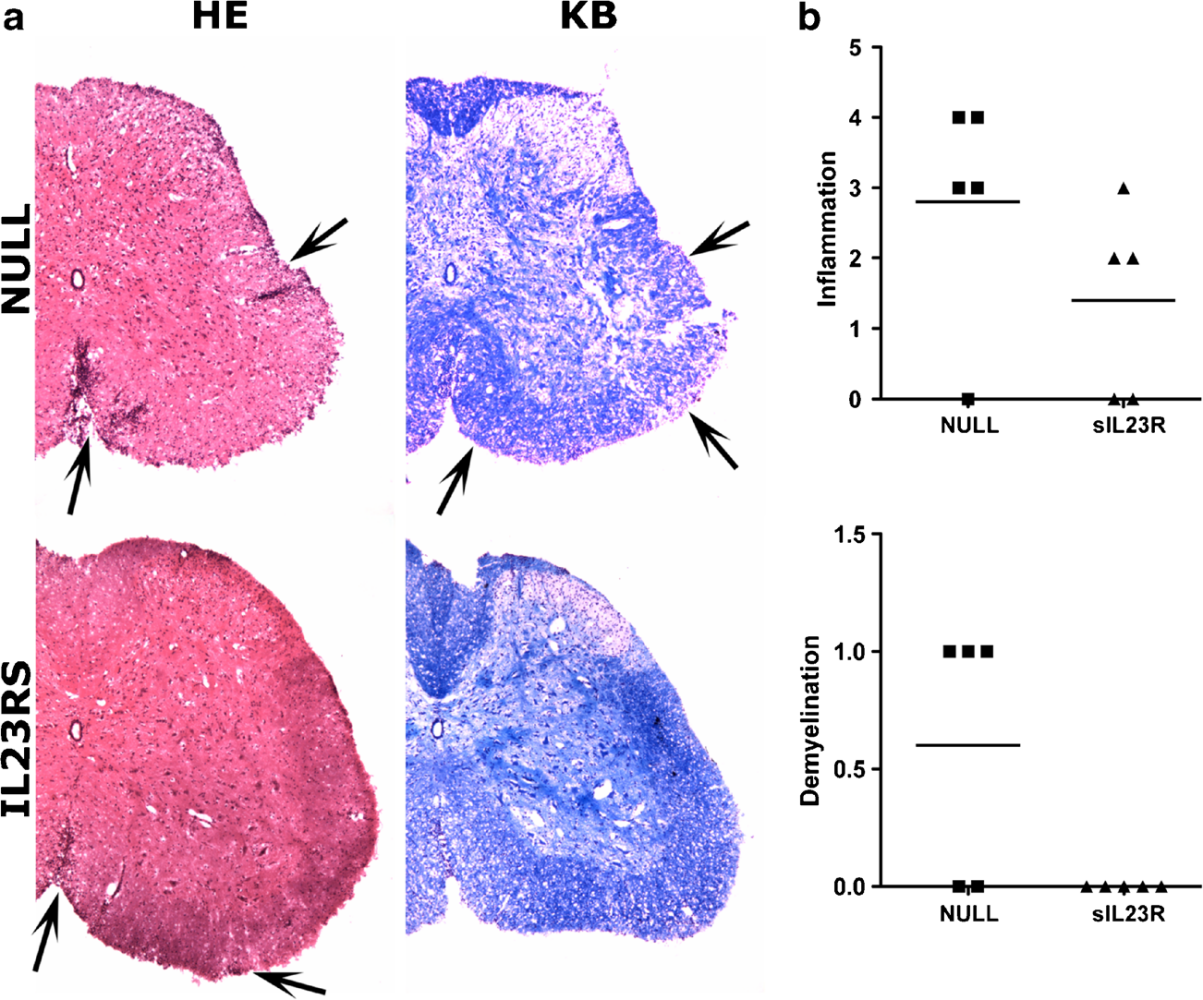
Inflammation [hematoxylin and eosin (HE)] and demyelination [Klüver–Barrera (KB)] in the spinal cord at day 14 postimmunization. (A) Representative sections (*n* = 5 mice per group). In HE staining arrows indicate inflammatory infiltrates. In KB staining arrows indicate areas of demyelination. (B) Inflammatory and demyelination score for null and soluble interleukin-23 receptor (sIL-23R) groups NULL = spinal cord of adeno-associated virus AAV8-null treated myelin oligodendrocytes glycoprotein (MOG) peptide 40–55 (MOG_40–55_)-immunized mice (*n* = 5); sIL-23R = spinal cord of AAV8–sIL-23R-treated MOG_40–55_-immunized mice (*n* = 5)

**Fig. 7:**
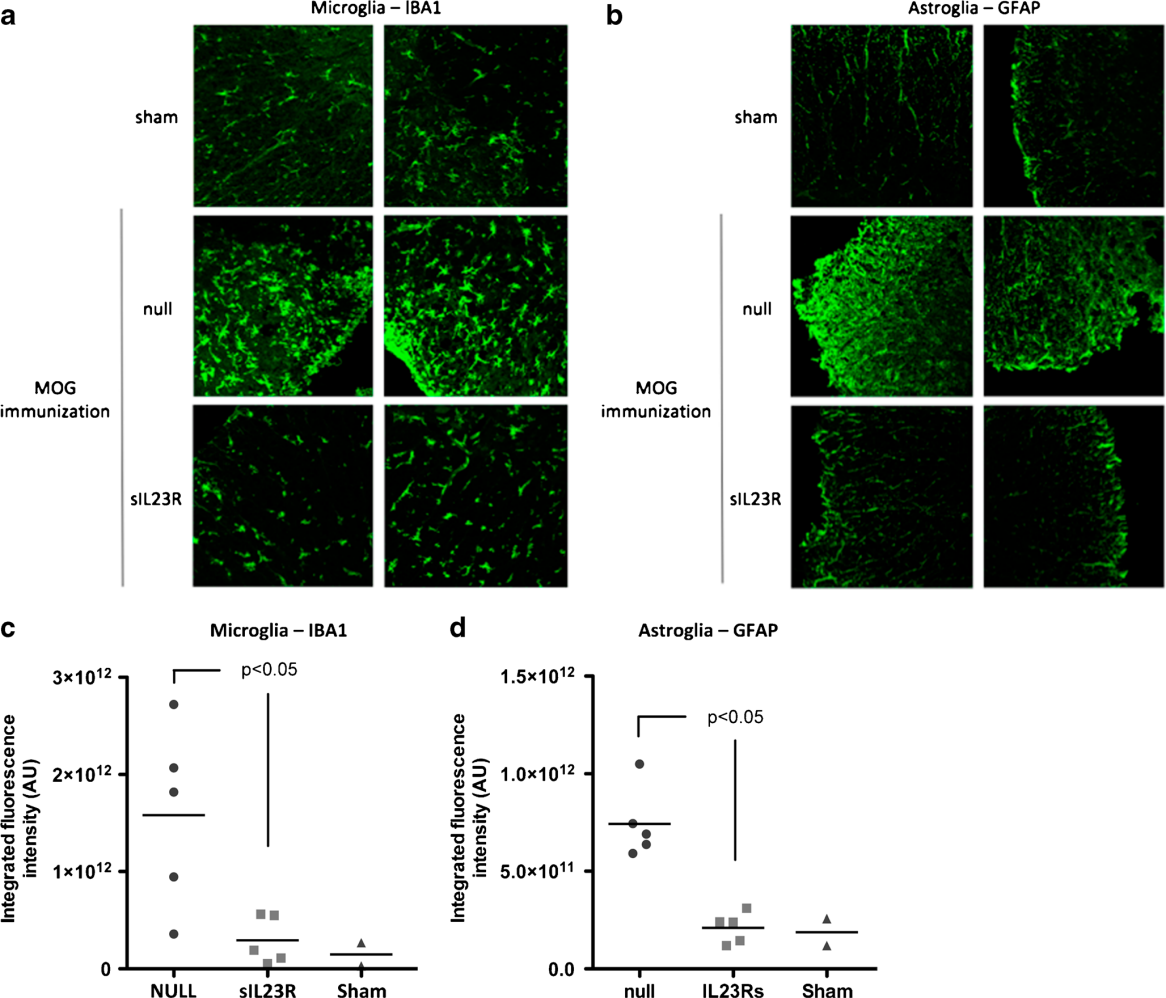
Representative pictures of (A) microglia and (B) astroglia activation in the spinal cord at day 14 postimmunization (*n* = 5 mice per group). Values of integrated fluorescence intensity following immunohistochemistry with specific markers (C) Iba1 (microglia) and (D) glial fibrillary acidic protein (astroglia). Values of integrated fluorescence intensity were determined in 5 mice per group, 12 slices per mouse, and 3 fields per slice (40× magnification) Sham = spinal cord of mice immunized without antigen; NULL = spinal cord of adeno-associated virus AAV8-null treated myelin oligodendrocytes glycoprotein (MOG) peptide 40–55 (MOG_40–55_)-immunized mice; sIL-23R: spinal cord of AAV8–soluble interleukin-23 receptor (sIL-23R)-treated MOG_40–55_-immunized mice.

A histopathological study was performed to assess the degree of inflammation and demyelination of the spinal cords of the treated animals. Of note, the histopathology of sIL-23R-treated mice was consistent with the observed clinical outcome. In the null-treated mice, demyelination and abundant inflammatory infiltrates (mainly composed of lymphocytes and macrophages) were observed in the white matter of the spinal cord. In contrast, in sIL-23R-treated mice, the inflammatory infiltrate was much more discrete and no demyelination was observed at day 14 p.i. (Fig. 6A, B). In agreement with this, microglial activation in sIL-23R-treated mice was similar than in sham-immunized mice, and astroglial activation was significantly lower than that observed in null-treated animals. For microglia activation, mean integrated fluorescence intensity (relative units) in null-treated mice was 1.6 × 10^12^ ± 9.3 × 10^11^ and in sIL-23R-treated mice it was 0.3 × 10^12^ ± 2.5 × 10^11^ (*p* = 0.03; Fig. 7A, C); and for astroglial activation mean integrated fluorescence intensity (relative units) in null-treated mice was 7.4 × 10^11^ ± 1.8 × 10^11^ and in sIL-23R-treated mice it was 2.1 × 10^11^ ± 7.8 × 10^10^ (*p* = 0.01; Fig. 7B, D).

## Discussion

MS is a neurodegenerative disease in which the Th17 immune response has a relevant role in maintaining and amplifying the autoreactive T lymphocytes against myelin, which eventually leads to demyelination in the CNS and severe neurological disability. Recent reports demonstrate that the activity of IL-23 is key in expanding and maintaining the Th17 cell population [26–29]. Therefore, blocking the IL-23/IL-23R interaction may inhibit the IL-23R-mediated signaling cascade and thus may have a therapeutic effect either at the initiation phase or during progression of disease.

It has been described that a therapy based on blocking IL-23 signaling by anti-p19 monoclonal antibodies prevented the induction of EAE and reversed the disease once established. Thus, Chen et al*.* [7] compared the anti-p40 therapy with anti-p19 therapy, concluding that although both approaches provided resistance to autoimmune inflammation, the first caused numerous side effects owing to the inhibition of both Th1 and Th17 pathways. Unfortunately, repeated administration of such biologicals, as the anti-α4 integrin antibody (natalizumab), appears to induce the formation of neutralizing antibodies against them with consequent loss of efficiency [30, 31]. To avoid these problems, Guo et al. [17] administered the soluble recombinant IL-23R cytokine-binding homology region to block the interaction between IL-23 and IL-23R, observing that their continuous administration significantly ameliorated clinical signs in the EAE model. In order to avoid continuous administration of the therapeutic molecule (either exogenous antibodies or recombinant IL-23R) we decided to study the therapeutic potential of an alternative approach based on the single administration of AAV vectors carrying sIL-23R. This approach would avoid continuous administration of the therapeutic molecule, while still selectively inhibiting the effects of IL-23 and thereby would specifically immunomodulate the Th17 pathway.

To this end, we designed the sIL-23R mouse gene *de novo*, based on a sIL-23R isoform described previously [24], and then generated AAV vectors carrying either the IL-23R or sIL-23R genes. Cells infected with the adenovirus vectors produced and secreted sIL-23R to the extracellular medium. Moreover, in infected cells, IL-23R was located at the cell membrane, whereas sIL-23R was observed in the cytoplasm.

Intravenous administration of AAV8–sIL-23R was associated with a delay in the onset of the first clinical signs, as well as a significant clinical improvement until the end of the experiment. Moreover, the variation in body weight was consistent with the clinical effect since EAE generally correlates with weight loss [32], and it was lower in the sIL-23R-treated mice than in the control-treated mice. Similarly, histopathological findings were also in accordance with the clinical improvement observed. Thus, in the spinal cord of sIL-23R-treated mice, reduced demyelination and the inflammatory component was observed. At the same time, both the astrocyte response and microglia activation were significantly lower in the treated animals, and similar to the control group immunized with saline that did not develop the disease. These results are in agreement with those reported by Guo et al [17]. Of note, since intravenous administration of AAV8 vectors at the indicated doses infect efficiently liver and skeletal muscle but not CNS [33, 34], the clinical effects observed must be due to systemic production of sIL-23R, thus hindering local high levels of sIL-23R being reached in the brain.

Interestingly, Yu and Gallagher [25] showed that, *in vitro*, human sIL-23R modulates the Th17 cells by inhibiting the phosphorylation of STAT3 caused by IL-23. Similarly, our studies also show that sIL-23R inhibits the phosphorylation of STAT3, highlighting the therapeutic potential of sIL-23R to antagonize IL-23-mediated STAT3 response.

However, when analyzing the medium of cultured splenocytes from mice treated with AAV8–sIL-23R, we found that only the levels of IFN-γ were statistically significantly different (3 times higher) than those observed in control-treated animals. This is consistent with data reported by other authors demonstrating that increased IFN-γ is associated with a better clinical outcome in EAE mice. In fact, IFN-γ is described as a potent negative regulator of the immune responses mediated by the IL-23/IL-17 axis that strongly inhibits the development of Th17 cells [35, 36]. Moreover, it has been observed that administration of anti-IFN-γ causes exacerbation of EAE in mice, while administration of exogenous IFN-γ in mice generated a protective effect against EAE [37–39]. In addition, IFN-γ-deficient and IFN-γ receptor-deficient mice show a more severe EAE [40, 41]. Therefore, the apparent clinical improvement of sIL-23R-treated mice matches the significant increase in IFN-γ production. However, this increase in IFN-γ was not accompanied by a significant decrease in the concentration of proinflammatory Th17 cytokines, such as IL-22 or IL-17A. In fact, even though a slight increase in granulocyte macrophage colony-stimulating factor levels was observed, no changes were detected in the pattern of the regulatory or anti-inflammatory cytokines, such as IL-10 and IL-4. Unfortunately, these results do not clarify the mechanism of action of this approach, although this will be extensively studied in future experiments, together with flow cytometric characterization of circulating and intra-CNS inflammatory cells.

Recently, there has been a major concern regarding the occurrence of serious side effects in current experimental treatments for MS [42, 43]. There have been > 200 cases of progressive multifocal leukoencephalopathy in patients treated with natalizumab (approximately 1–11/1000 cases, depending on other risk factors and drug exposure time) [44]. However, no studies on the side effects associated with long-term specific blockage of the IL-23/IL-23R interaction are known. In this regard, long-term studies with tildrakizumab will help to address this issue.

In summary, the use of vectors carrying sIL-23R for the treatment of MS may be a new therapeutic strategy in the current scenario where most of the current therapies and clinical trials include the use of antibodies or recombinant proteins. Unfortunately, in the EAE model, a very strong immune response after sensitization with myelin antigen prevents subsequent administration of viral vectors. In order to study the efficiency of the gene therapy treatment when given after onset of EAE, sIL-23R constructs carrying inducible promoters, or the use of different EAE models must be tested. Moreover, it would be interesting to test the effectiveness of this strategy in other autoimmune diseases in which the Th17 pathway plays a key role, such as psoriasis, Crohn’s disease or rheumatoid arthritis.

## Electronic supplementary material

Below is the link to the electronic supplementary material.

ESM 1 Required Author Forms Disclosure forms provided by the authors are available with the online version of this article (PDF 1224 kb)

ESM 2 Required Author Forms Disclosure forms provided by the authors are available with the online version of this article (PDF 1225 kb)
